# Entrapped surgical needle In the valveless AirSeal trocar: A case report and literature review

**DOI:** 10.1016/j.eucr.2024.102826

**Published:** 2024-08-14

**Authors:** Ibrahim Can Aykanat, Mevlana Derya Balbay

**Affiliations:** a Koc University Hospital, Urology Clinic, Istanbul, Turkey; b Koc University School of Medicine, Department of Urology, Istanbul, Turkey; c VKV American Hospital, Urology Clinic, Istanbul, Turkey

**Keywords:** AirSeal trocar, Robot-assisted laparoscopic surgery, Surgical needle retention, Valveless trocar

## Abstract

**Background:**

Valveless trocars like AirSeal system are maintain a stable pneumoperitoneum and reduce instrument friction.

**Case presentation:**

A 65-year-old man's robotic radical cystectomy was complicated by a missing needle while using AirSeal system. The needle was detected via backward inspection using the endo camera inserted through the trocar, tip at its most distal end let the visualization of the needle within the air channels and confirmed with a trocar X-ray.

**Conclusion:**

Our findings suggest that retrograde inspection and targeted radiography of the trocar, prior to patient imaging, can be helpful in locating the lost needle and prevent prolongation of surgeries.

## 1 Introduction and background

Surgical equipment, such as sutures and clips used in laparoscopic and robot-assisted laparoscopic surgeries, is typically delivered to the surgical field through assistant trocars. In conventional valved trocars, it is common for surgical equipment to become stuck in the valve system during removal from the surgical field. However, in new-generation valveless trocars, the lack of a valve mechanism reduces the retention of equipment inside the trocar. We present our case report describing a retained surgical needle incident in the valveless mechanism of the AirSeal (AirSeal®, ConMed, Utica, NY, USA) assistant trocar port during robot-assisted laparoscopic radical cystectomy and suggest recommendations on intraoperative management of a missing needle during laparoscopic or robotic surgical procedures.

## 2 Presentation of case

**Fig. 1:**
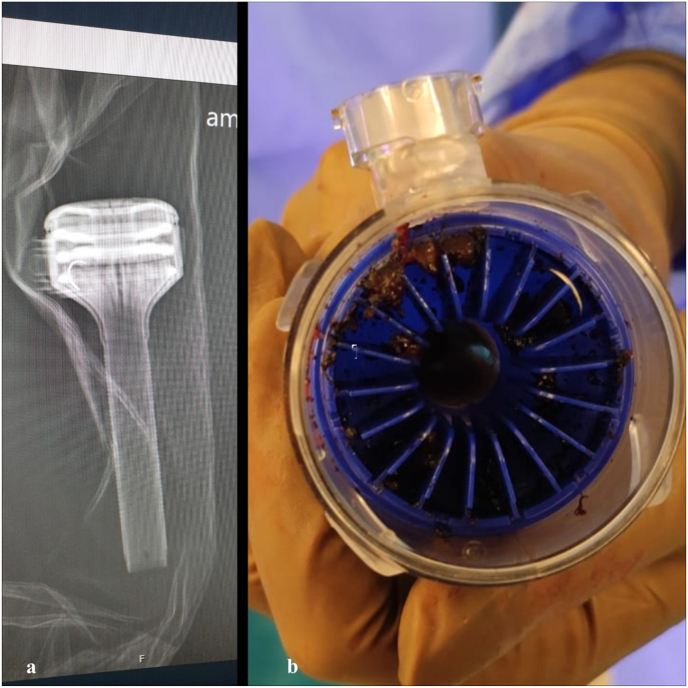
**A.** The appearance of a needle stuck inside an Airseal trocar under x-ray radiography. **1b.** The image of a needle stuck in the air channels of a disassembled Airseal trocar.

A 65-year-old male patient presented with pT2 high-grade urothelial carcinoma was scheduled for robot assisted endopelvic fascia sparing radical cystoprostatectomy extended lymph node dissection and Balbay's pouch which is an intracorporeal orthotopic ileal neobladder with Antireflux mechanism performed by utilizing the da Vinci Xi Surgical System (Intuitive Surgical, Sunnyvale, CA).^1^^,^^2^ Four trocars for robotic arms and one 12 mm AirSeal trocar were placed on the abdomen. During the surgery, 17 mm RB-1 needle on a 3-0 Stratafix® (Ethicon) suture was removed through AirSeal trocar with laparoscopic needle driver with a slight resistance. Upon extraction of the suture, no needle was observed on the suture outside of the trocar. The trocars air cap filter was initially examined, along with the semi-transparent plastic surfaces. Later the robotic zero-degree camera was retrieved and inserted in through the AirSeal trocar and inspected antegradely. Next abdominal cavity and intestines were examined thoroughly laparoscopically. With none of these manoeuvres lost needle could be found. Lastly, endo camera was retrogradely inserted through the distal end opening of the AirSeal trocar. At this inspection, the entrapped needle was visualized inside the peripherally located air channels (Video 1). AirSeal trocar was removed and examined with X-ray imaging (Fig. 1a) which showed the needle inside the air channel of the trocar. The trocar was disassembled, and then needle was removed (Fig. 1b). Subsequently, the trocar was reassembled, and the remaining part of the surgery continued uneventfully.

Supplementary video related to this article can be found at https://doi.org/10.1016/j.eucr.2024.102826

The following is the supplementary data related to this article:

Video S1 1 Video S1

## 3 Discussion and literature review

**Fig. 2:**
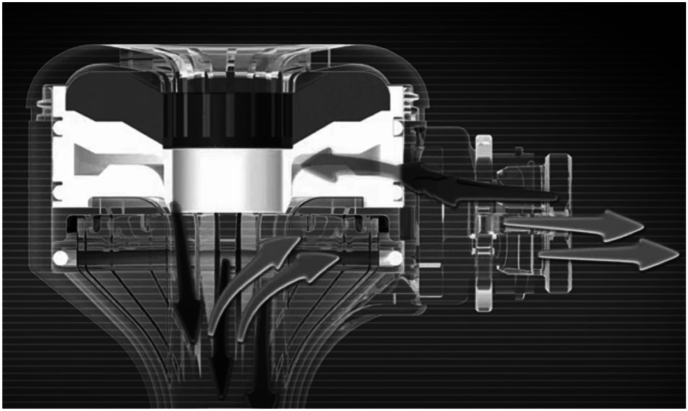
An illustration of the Airseal trocar showing the airflow.

The advantages of using the AirSeal system include providing a stable pneumoperitoneum even during aspiration, continuous suction of surgical smoke, reduced friction between trocars and surgical instruments and prevent any smudge on the lens due to the valveless mechanism. AirSeal systems utilize high-flow CO2 injection through the centrum of the working channel and smoke was evacuated through the periphery of the trocar to create a pressure barrier which sustains the pneumoperitoneum. The pressure of the pneumoperitoneum is measured at the distal tip of the trocar, while gas from the proximal side is extracted, filtered, and reintroduced through the working channel.^3^ In this case, we believe that after the needle was detached from the suture, it was entrapped in the outward airflow path, in the air channels of the trocar (Fig. 2). By this means, detached needle was not thrown into the abdominal cavity but instead entrapped within the AirSeal trocar. We believe that this is another advantage of AirSeal.

To the best of our knowledge, two cases have been reported in the English literature where the needle detached from the suture and became entrapped within the AirSeal trocar. In the first of these cases, Moynihan and Moinzadeh observed the detached needle within the semi-transparent plastic of the inner aspect of the AirSeal trocar, separate from the working lumen.^4^ In the second case reported by McClintock et al., the needle entrapped within the AirSeal was neither visible within the semi-transparent plastic of the inner aspect of the AirSeal trocar, nor could be found in the abdomen. Intraoperative X-ray radiography revealed the needle inside the trocar.^5^

A missing needle imposes anxiety on the surgeon. Before going to detailed examination of the abdominal cavity we recommend to quickly remove the trocar's cap and valve system and inspect its interior with a camera. Then, before going to inspection of abdominal contents and cavity, a retrograde visual examination of the AirSeal trocar should be conducted in addition to careful outside environmental area for the possibility of the needle falling out of the trocar. X-ray radiography should be performed including Air Seal trocar in addition to the surgical field after laparoscopic abdominal control.

In our case we found the missing needle after retrograde inside examination of the Air Seal trocar through its distal end. Subsequently, we removed the AirSeal trocar and solely performed X-ray radiography of the trocar to confirm its place. Thanks to this procedure, the need to undock the robot and adjust the Trendelenburg position was omitted for X-ray examination of the patient, since specially in robot-assisted laparoscopic surgeries, due to the patient's position and the presence of the robot is not suitable for quick X-ray examination. We believe that removing the trocar and examining it radiographically would save time and effort, even if the needle is not visualized on retrograde internal inspection of the trocar.

## 4 Conclusion

In surgical cases of lost needle incidents where valveless trocars like AirSeal is used, the possibility of the needle entering the internal air channels due to air circulation should be kept in mind. Retrograde inspection into the trocar can facilitate needle detection. Considering the internal structure of the AirSeal trocar in such situations, taking X-ray radiographs of the trocar before the patient can prevent time and effort loss.

## Institutional reviewer board and the approval number

N/A.

## Informed consent

Written consent to publish this report was obtained from the study participant.

## Registry and the registration no. of the study/trial

N/A.

## CRediT authorship contribution statement

**Ibrahim Can Aykanat:** Writing – original draft. **Mevlana Derya Balbay:** Writing – review & editing.

## Declaration of competing interest

The authors declare no conflict of interest.
